# A Lethal Pseudofilovirus Model in *Ifnar1*(-/-) Mice Using Recombinant Vesicular Stomatitis Viruses

**DOI:** 10.3390/v18080878

**Published:** 2026-08-11

**Authors:** Anna V. Mamatkulova, Inna V. Shuliakova, Olga V. Zubkova, Dmitrii A. Reshetnikov, Anna A. Iliukhina, Ilya D. Zorkov, Daria M. Grousova, Daria M. Savina, Olga Popova, Denis I. Zrelkin, Polina P. Goldovskaya, Ekaterina G. Samokhvalova, Valentin V. Azizyan, Dmitry V. Shcheblyakov, Boris S. Naroditsky, Alexander L. Gintsburg, Denis Yu. Logunov

**Affiliations:** 1 National Research Center for Epidemiology and Microbiology Named After Honorary Academician N. F. Gamaleya, Ministry of Health, Moscow 123098, Russia; 2 P. Hertsen Moscow Oncology Research Institute-Branch of the National Medical Research Radiology Center, Ministry of Health, Moscow 119121, Russia; 3 Department of Infectology and Virology, I. M. Sechenov First Moscow State Medical University (Sechenov University), Ministry of Health, Moscow 119435, Russia

**Keywords:** rVSV surrogate model, rVSV-EBOV, filovirus infection model, Ebola virus, Marburg virus, Sudan virus, Bundibugyo virus, *Ifnar1*-knockout mice

## Abstract

Filoviruses cause highly lethal infectious diseases with hemorrhagic symptoms and extremely high fatality rate (up to 90%). Although two vaccines against Ebola virus (EBOV) are licensed for application in endemic areas, vaccines against other filoviruses (SUDV, BDBV, MARV) are still at pre-clinical or clinical stages. The development of vaccines requires protective efficacy studies using animal models of infection. However, animal studies using wild-type filoviruses require maximum biosafety level (BSL-4), which hinders filoviral vaccine advancement. In this study, we developed a surrogate animal model of EBOV-, SUDV-, BDBV- and MARV- GP-mediated entry using *Ifnar1*-knockout (-/-) mice and recombinant vesicular stomatitis viruses (rVSVs), carrying a genetic sequence of filoviral glycoprotein (GP) and pseudotyped with correspondent GP. We showed that rVSV-EBOV-GP was 100% lethal for *Ifnar1*(-/-) mice after inoculation by multiple routes, and its active propagation was within 24 h post-infection. We performed dose-dependent lethality assessment in small groups to estimate median lethal doses for rVSV-EBOV-GP, rVSV-SUDV-GP, rVSV-BDBV-GP, rVSV-MARV-GP in *Ifnar1*-knockout mice and showed high viral load in liver and immune cell reservoirs. The developed models contribute to safe and effective research of filoviral vaccines and therapeutic antibodies under BSL-2 conditions.

## 1. Introduction

Ebola and Marburg viruses are members of the Filoviridae family and cause highly fatal infectious diseases: Ebola virus disease (EVD) and Marburg virus disease (MVD). Three of six species of Ebola virus are highly pathogenic for humans and cause EVD: Ebola virus (EBOV, formerly Zaire), Sudan virus (SUDV), and Bundibugyo virus (BDBV) [1]. Since the discovery of filoviruses in the 1960s and 1970s, more than 40 EVD outbreaks and nearly 20 MVD outbreaks have been recorded mainly in Central and West Africa [2,3]. The largest EVD outbreak led to the epidemic in West Africa in 2014 to 2016, resulting in more than 28,000 cases and over 11,000 deaths [4]. The scale of the 2014–2016 EVD epidemic prompted the global community to accelerate the development of vaccines against Ebola virus disease.

In recent years, filovirus outbreaks have regularly occurred in endemic and new areas. In November 2022, the first outbreak in the last ten years of EVD Sudan was reported in Uganda, with 164 cases and 55 deaths. During EVD outbreak in the Democratic Republic of the Congo (DRC) in September–December 2025, 64 confirmed cases and 45 deaths were reported [2]. In 2023, two MVD outbreaks in Equatorial Guinea and Tanzania were reported; both were the country’s first outbreaks of the disease. In September–December 2024, an outbreak of MVD was declared for the first time in Rwanda, with 66 cases and 15 deaths reported. In January–March 2025, a Marburg virus disease outbreak occurred in Tanzania, resulting in ten deaths [3]. A recent outbreak of Bundibugyo virus in DRC was declared a public health emergency of international concern (PHEIC) on 16 May 2026 [5]. In this way, filovirus outbreaks are sporadic, with unpredictable area expansion, which can quickly escalate into epidemics and lead to exported cases. Case fatality rates for filovirus infections reach up to 90% [6]. The World Health Organization (WHO) has included filovirus diseases in the WHO R&D Blueprint Priority Pathogens [7].

To date, two vaccines against EBOV have been approved by international health regulators and have already been used in endemic regions during recent outbreaks [8,9,10]. Nevertheless, no licensed vaccines are currently available for Sudan virus disease, Bundibugyo virus disease, or Marburg virus disease. To anticipate future filovirus outbreaks, the WHO has defined a target product profile (TPP) for filovirus candidate vaccines, which includes recommendations for an expanded antigenic composition [11]. Active research is ongoing to develop multivalent filovirus vaccines.

An essential step in vaccine development is in vivo evaluation of protection. However, such studies using wild-type Ebola and Marburg viruses require BSL-4 conditions and are inaccessible to most research institutes. Non-human primates (NHPs) are most commonly used in filoviral infection models, but their maintenance is highly labor-intensive and costly [12]. Rodents can also serve as filovirus infection models. Researchers typically employ virus strains adapted to the used species, but this requires time-and-labor-consuming passaging of wild-type filoviruses in animals in BSL-4 conditions. Also, most of the established models focus on EBOV infection, whereas models for SUDV, BDBV and Marburg virus (MARV) infections are less commonly represented in such studies. Collectively, the complexity of protective efficacy studies continues to be a major factor constraining the development of effective filovirus vaccines and therapeutics.

To make filoviral vaccine research more accessible, surrogate animal models of filovirus infection using recombinant viral vectors (mainly rVSVs) were developed, enabling studies to be performed under BSL-2 instead of BSL-4 conditions. A number of surrogate rVSV-EBOV models using Syrian hamsters and transgenic mice have already been established. In our study, we aimed to expand such BSL-2-compatible models to other filoviral infections (SUDV, BDBV, MARV) using pseudotyped recombinant VSVs and provide their detailed characteristics to enhance reproducibility in animal studies. We used transgenic mice with an *Ifnar1*-gene knockout (-/-) as test subjects. The lack of interferon receptor type I function leads to disruption of the signaling cascades associated with the antiviral and immunomodulatory response. This increases host susceptibility to viral infections and creates permissive environment for uncontrolled replication of recombinant viral vectors, including vaccine vectors, normally safe in immunocompetent animals. A uniform surrogate model for in vivo studies of GP-mediated entry would significantly simplify the testing of multivalent filoviral vaccines that comply with the WHO strategy [11].

## 2. Materials and Methods

### 2.1. Ethical Statement

All animal experiments were approved by the local animal ethics committee (protocol #86, 23 December 2024) and performed in compliance with the Guide for the Care and Use of Laboratory Animals (NIH Publication #85–23, revised 1996) and the National Standard of the Russian Federation.

### 2.2. Animals

Transgenic knockout mice C57BL/6NCya-ifnar1em1/Cya were purchased from Cyagen (Cat# C001268). The strain represents a model of *Ifnar1* knockout (KO), constructed by knocking out exon 2 (E2) of the mouse *Ifnar1* gene. This modification leads to a deficiency in the function of type I IFN receptors, resulting in a diminished immune response, heightened susceptibility to viral infections, increased levels of myeloid lineage cells in peripheral blood and bone marrow, and a decreased immune response to immunostimulatory DNA. The *Ifnar1*-knockout (-/-) mice were bred at an onsite animal breeding facility under SPF conditions and 5–6-week-old mice were subsequently transferred to BSL-2 conditions. The animals were housed in ventilated ISOCage N system (Techniplast, Buguggiate, Italy) with free access to autoclaved drinking water and standard chow diet.

### 2.3. Viruses and Cell Lines

Recombinant vesicular stomatitis viruses, pseudotyped with filoviral glycoproteins and carrying the corresponding GP gene sequences (rVSV-Filo-GP) were constructed and assembled in Gamaleya NRCEM. The gene of native VSV glycoprotein G was deleted and replaced with the gene of the full-length filoviral glycoprotein, as a result, recombinant viral particles were pseudotyped with solely filoviral GP. rVSV-Filo-GP vectors were constructed as follows: nucleotide sequences of EBOV, SUDV, BDBV, MARV full-length GP were cloned into pVSV-ΔG plasmid. For viral recovery of rVSVs, BHK21 cells were seeded on a 6-well plate (3 × 10^5^ cells/well), co-transfected with plasmids carrying the following genes: DNA-dependent RNA polymerase of bacteriophage T7 (1 μg/well), N protein (1 μg/well), P protein (0.5 μg/well), L protein (0.2 μg/well) and each obtained genetic construct rVSV-Filo-GP, containing full VSV genome (ΔG) and gene of filoviral GP (2 μg/well). Transfection was performed using the Lipofectamine-2000 reagent (Invitrogen, Waltham, MA, USA) according to the manufacturer’s instructions. Ninety-six hours after transfection, the culture medium was collected and transferred to Vero E6 cells for reinfection (6-well plate, 8 × 10^5^ cells/well). The production of recombinant viruses was detected by the presence of cytopathic effect and confirmed by qRT-PCR. Four rVSVs were constructed and used to establish GP-mediated entry models for EBOV, SUDV, BDBV and MARV, correspondingly: rVSV-EBOV-GP, rVSV-SUDV-GP, rVSV-BDBV-GP, rVSV-MARV-GP. rVSVs propagation was performed in Vero E6 cells in DMEM (HyClone Cytiva, Vienna, Austria) supplemented with 2% heat-inactivated FBS (Capricorn Scientific GmbH, Dreihausen, Germany), L-glutamine (4 mM) and penicillin/streptomycin solution (100 IU/mL; 100 μg/mL) (PanEco, Moscow, Russia). The cells were infected at multiplicity of infection (MOI) = 0.01 and incubated at 37 °C in 5% CO_2_. The culture medium was collected at 48 h and clarified via centrifugation at 10,000× *g* for 10 min at +4 °C, then aliquoted and stored at −80 °C.

### 2.4. Plaque Assay

Infectious titers of rVSV-Filo-GP were determined by plaque-forming assay. The day prior to the assay Vero E6 cells (ATCC CRL-1586, ATCC, Manassas, VA, USA) were seeded in 24-well plates. The next day serial tenfold dilutions of rVSV samples in DMEM with 2% heat-inactivated FBS were prepared and immediately transferred to cell monolayer in triplicates (100 μL per well). The plates were incubated at 37 °C in 5% CO_2_ for 1 h. Then, rVSV-containing medium was discarded and cells were overlayed with pre-heated mixture of DMEM-CMC (DMEM with 2% heat-inactivated FBS mixed with carboxymethyl cellulose, 0.7%). The plates were incubated at 37 °C in 5% CO_2_ for 48 h. After incubation DMEM-CMC overlay was aspirated, the cells were gently washed with sterile isotonic saline solution, fixed with 10% formaldehyde for 30 min, stained with 1% crystal violet in 20% ethanol for 15 min, then washed with dH_2_0. The plaques were counted visually, using negatoscope (or binocular for abundant amount of the plaques).

### 2.5. PCR

Viral RNA was isolated using TriZol reagent according to the manufacturer’s instructions. Complementary DNA (cDNA) was synthesized using HiScript III 1st Strand cDNA Synthesis Kit (Vazyme Biotech Co., Ltd., Nanjing, China) according to the manufacturer’s protocol. Amplification and detection of target fragments of viral RNA by qPCR were performed using primers targeting a fragment of the VSV N-protein gene: forward 5′-AAACATCGGGAAAGCAGGG-3′ (100 μM), reverse 5′-CAACCATTTGTCATCTGCGC-3′ (100 μM), and probe 5′(FAM)-ACCTTGTATCCTTGAAAGCCCTGGACGG-(BHQ1)-3′, (100 μM). The amplification was carried out using the following program: +95 °C 180 s; (+95 °C 15 s, +60 °C 30 s, +72 °C 20 s (read)) × 40 cycles, with Bio-Rad CFX-96 Real-Time PCR Detection System (Bio-Rad Laboratories, Hercules, CA, USA).

### 2.6. Western Blotting

To confirm GP expression in vitro, rVSV-Filo-GP preparations were added to Vero E6 cell monolayer in T25 flasks at 0.01 MOI, and 48 h later the cells were collected and lyzed with M-PER™ Mammalian Protein Extraction Reagent (Thermo Scientific, Waltham, MA, USA), according to the manufacturer’s protocol. The lysates were subsequently denatured in Laemmli buffer at 95 °C for 10 min. For the electrophoresis, precast 4–20% Mini-PROTEAN^®^ TGX™ Stain-Free™ gels (Bio-Rad, Hercules, CA, USA) Precision Plus Protein™ Dual Color molecular weight standards (Bio-Rad, USA), and TGS (tris-glycine-SDS) buffer were used. Proteins separated on the gel were transferred to nitrocellulose membrane with a 0.2 µm pore size (Bio-Rad, USA) in a Trans-Blot Turbo transfer system (Bio-Rad, USA). The membrane was blocked in 5% skim milk in TPBS buffer (0.1% Tween-containing phosphate saline buffer) at 37 °C for 1 h at 300 rpm using an orbital shaker. Subsequently, primary and secondary antibodies were incubated in the same conditions. The membranes were 5x washed in TPBS buffer after antibody incubations. The following filovirus glycoprotein-specific antibodies were used: Mouse anti-Ebola Virus GP mAb (4F3) (0201-020; IBT bioservices, Rockville, MD, USA), Ebola virus EBOV (Subtype Sudan, strain Gulu) Glycoprotein/GP1 (mucin domain deleted) Antibody, Rabbit MAb (40094-R106; Sino Biological, Beijing, China), Ebola virus EBOV (subtype Bundibugyo, strain Uganda 2007) GP1/Glycoprotein Antibody, Rabbit PAb (40368-T46; Sino Biological, China), and Mouse anti-MARV mAb (2D8) (0203-025; IBT bioservices, USA). As secondary antibodies, horseradish peroxidase-conjugated goat anti-mouse IgG H&L (preadsorbed; ab97040; Abcam, Cambridge, UK) and donkey anti-rabbit IgG H&L (HRP) (ab6802; Abcam, UK) were used. Detection was performed by chemiluminescence using Clarity™ Western ECL Substrate (Bio-Rad, USA).

### 2.7. Mouse Challenge and Sampling

*Ifnar1*(-/-) transgenic male mice were used in all experiments. For choice-of-route studies, groups of mice (n = 3) received intraperitoneal (i.p.), intramuscular (i.m.), intranasal (i.n.), or intragastric (i.g., using oral gavage) injection of 10^4^ plaque-forming units (PFUs) of rVSV-EBOV-GP per animal, and the control groups received the same volume of sterile isotonic saline solution. The mice were checked for survival, weight loss and clinical symptoms until day 14. Clinical scoring categories included body posture (0–3), activity (0–3), coat condition (0–3), and weight loss (0–3). Total score was calculated as the sum of the scores in four categories; the cumulative humane endpoint score was 10. Body posture: 0—normal/straight, 1—slightly hunched, 2—hunched, 3—extremely hunched, prostrate/eyes closed. Activity: 0—active, 1—reduced activity, 2—changes in gait, such as sluggish and reluctant movement, or hyperactivity and excessively active movement, 3—severely impaired gait (e.g., stumbling, falling to one side) or significant restlessness, including tremors, vocalization, or aggression, humane endpoint—immobility, lying on side. Coat condition: 0—shiny, well-groomed hair coat, 1—unkempt/ruffled or dull, not shiny hair coat, 2—some indication of piloerection is present (i.e., ‘rough’ coat), but it is not obvious over the mouse’s entire surface area, 3—generalized piloerection, i.e., obvious, very rough coat over the majority of the body’s surface area. Weight loss: 0—change of 0–5%, 1—change of 5–10%, 2—change of 10–15%, 3—change of 15–20%, humane endpoint—change of 20% or more.

For viral load dynamic study, the mice received i.p. 10^4^ PFU of rVSV-EBOV-GP per animal. The control group (n = 3) was i.p. given sterile isotonic saline solution. The animals were euthanized 2 h, 24 h, 48 h post-infection (n = 5 at each time point) and the samples of 15 organs were obtained from each animal (oral swab, brain, thymus, lung, heart, blood, liver, spleen, stomach, pancreas, small intestine, large intestine, kidney, hind paw thigh muscle, inguinal lymph node). For rVSV-Filo-GP viral load studies, groups of mice (n = 5) received i.p. 10^4^ PFU of rVSV-EBOV-GP, rVSV-SUDV-GP, rVSV-BDBV-GP or rVSV-MARV-GP per animal. The control group (n = 3) was i.p. given sterile isotonic saline solution. The animals were euthanized 48 h after the challenge and the samples of the same 15 organs were obtained from each animal. For dose-dependent lethality assessment, groups of mice (n = 3) were i.p. infected with rVSVs at a tenfold range of doses: 10^6^ to 10^−3^ PFU per animal (roughly 10^10^–10^1^ genome copies per animal). The control groups (n = 3) were i.p. given sterile isotonic saline solution. After infection, body weight and survival were monitored for two weeks.

### 2.8. Viral Load Assay

The blood samples were collected in the tubes containing dry K3-EDTA, the oral swab was collected in 1 mL of sterile PBS, the solid organ samples were weighted and a corresponding volume of PBS (*w*/*w*) was added to prepare 10% homogenates, except 2% homogenate for the lymph node. The samples of solid organs were homogenized using MPbio FastPrep-24 (MP Biomedicals, Irvine, CA, USA) and centrifuged at 15,000× *g* for 10 min, and the supernatants were used for viral load assay. Infectious titer in the samples (TCID_50_/mL) was determined using a 50% tissue culture infectious dose (TCID_50_) assay on Vero E6 cells. The day prior to the assay, Vero E6 cells were seeded in 96-well plates to yield 90–100% confluency the next day (day of the assay). DMEM supplemented with 2% heat-inactivated FBS was used for sample dilution. The samples were titrated in 96-well plates in a total volume of 100 μL, with tenfold dilutions in four replicates and immediately transferred to the cell monolayer. The plates were incubated for 48 h at 37 °C in 5% CO_2_ and the cytopathic effect (CPE) of the rVSV was determined visually. The TCID_50_ titer was calculated according to the Spearman–Kaerber method [13]. The viral load results for the lymph node were subsequently recalculated to match 10% homogenates.

### 2.9. Statistical Analysis

Statistical assessment of the differences was performed using GraphPad Prism 9 software (v. 9.3.1, GraphPad, Boston, MA, USA). The normality of data distribution was analyzed using the Shapiro–Wilk test. For analysis of two groups, unpaired *t*-test or the Mann–Whitney test was used for normal and non-normal distributions, respectively. For analysis of multiple groups, one-way ANOVA or the Kruskal–Wallis test was used for normal and non-normal distributions, respectively. For pairwise comparisons, differences were considered significant at *p* < 0.05. For multiple comparisons, a Bonferroni correction was applied to adjust the significance level. Mean lethal doses (MLDs) of rVSVs in *Ifnar1*(-/-) mice were estimated using Spearman–Karber method

## 3. Results

**Figure 1:**
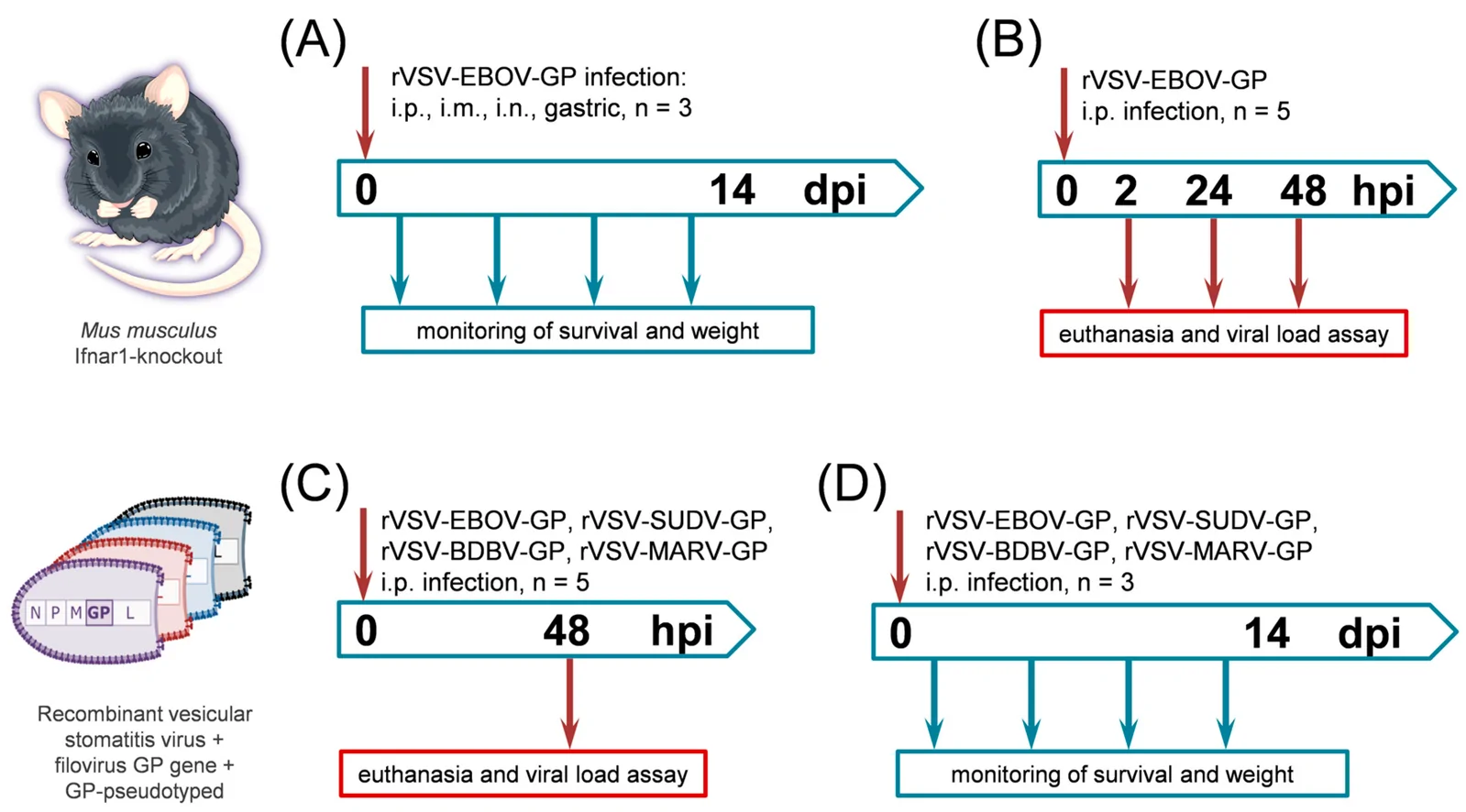
Study design. *Ifnar1*-knockout mice were infected with recombinant VSVs, carrying filovirus GP gene and pseudotyped with GP. (**A**) rVSV-EBOV-GP infection by various routes for survival and weight analysis; (**B**) viral load dynamics of rVSV-EBOV-GP i.p. infection; (**C**) endpoint viral load of rVSV-EBOV-GP, rVSV-SUDV-GP, rVSV-BDBV-GP, rVSV-MARV-GP i.p. infection; (**D**) dose-dependent lethality assessment of rVSV-EBOV-GP, rVSV-SUDV-GP, rVSV-BDBV-GP, rVSV-MARV-GP in *Ifnar1*(-/-) mice. For (**A**–**C**) experiments the mice were infected with 10^4^ PFU/animal. Viral load was analyzed in homogenates of the organs by TCID_50_ titration on Vero E6 cells; i.p.—intraperitoneal, i.m.—intramuscular, i.n.—intranasal, dpi—days post-infection, hpi—hours post-infection.

The first objective was to choose an optimal route to inoculate *Ifnar1*(-/-) mice, using rVSV-EBOV-GP as a model vector, and then to establish the propagation dynamics of rVSV-EBOV-GP in various organs of the mice after inoculation by the selected route. Then we estimated the approximate mean lethal doses of rVSV-EBOV-GP, rVSV-SUDV-GP, rVSV-BDBV-GP, rVSV-MARV-GP and determined endpoint viral titers (Figure 1).

### 3.1. Route-Dependent Lethality in Ifnar1-Knockout Mice After rVSV-EBOV-GP Infection

**Figure 2:**
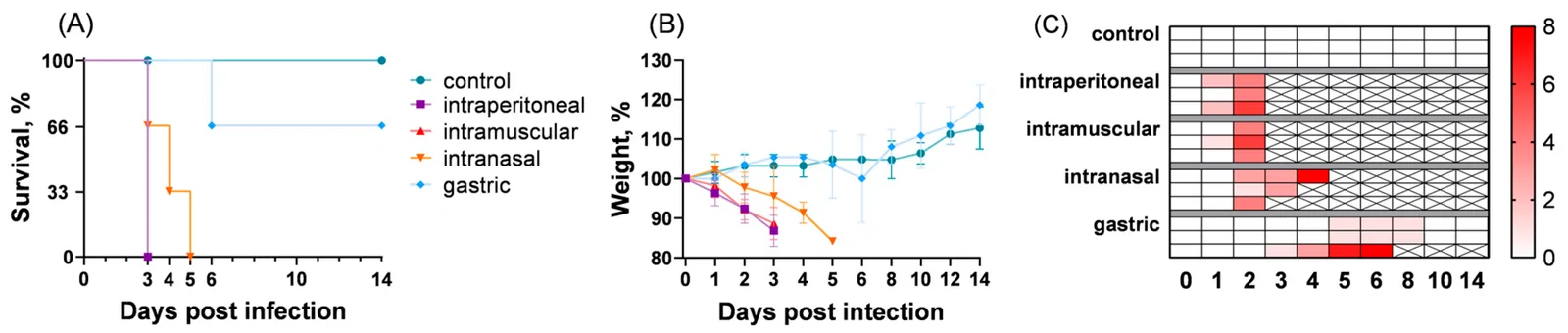
The susceptibility of *Ifnar1*-knockout mice to rVSV-EBOV-GP infection by various routes. Subfigures: (**A**) survival curves; (**B**) mean weight in all groups; (**C**) mean clinical score. The infectious dose was 10^4^ PFU per animal, n = 3 in all groups. Weight loss was measured as the percent weight loss compared with the initial weight on day 0. Clinical score included weight loss, activity level, appearance of coat and posture. The bars show standard deviation. The control group did not exhibit any clinical symptoms or experience any weight loss throughout the experiment.

The control group did not exhibit any clinical symptoms or experience any weight loss throughout the experiment. Intraperitoneal and intramuscular challenge led to 100% lethality at 3 dpi (days post-infection) (Figure 2A). Intranasal infection of rVSV-EBOV-GP was lethal for 1/3 of the mice at 3 dpi, and at 5 dpi all animals died. All mice in i.p., i.m., and i.n. groups showed clinical symptoms of infection (weight loss, hunching, rough coat and piloerection). After a gastric challenge of rVSV-EBOV-GP, 1/3 of the mice showed developing clinical symptoms and succumbed at 6 dpi, at the end of the observation period 2/3 animals (66%) survived and did not show weight loss or developing clinical symptoms—a slightly rough coat was observed at 3–8 dpi, but it resolved at 10 dpi in both animals (Figure 2B).

Intraperitoneal rVSV-Filo-GP challenge was selected for experimental consistency and reproducibility [14]. The endpoint of choice for viral load experiments was 48 h post-infection as day 2 was the last day with 100% survival rate in the intraperitoneal group.

### 3.2. Dose-Dependent Lethality Assessment of rVSV-Filo-GP in Ifnar1-Knockout Mice

**Figure 3:**
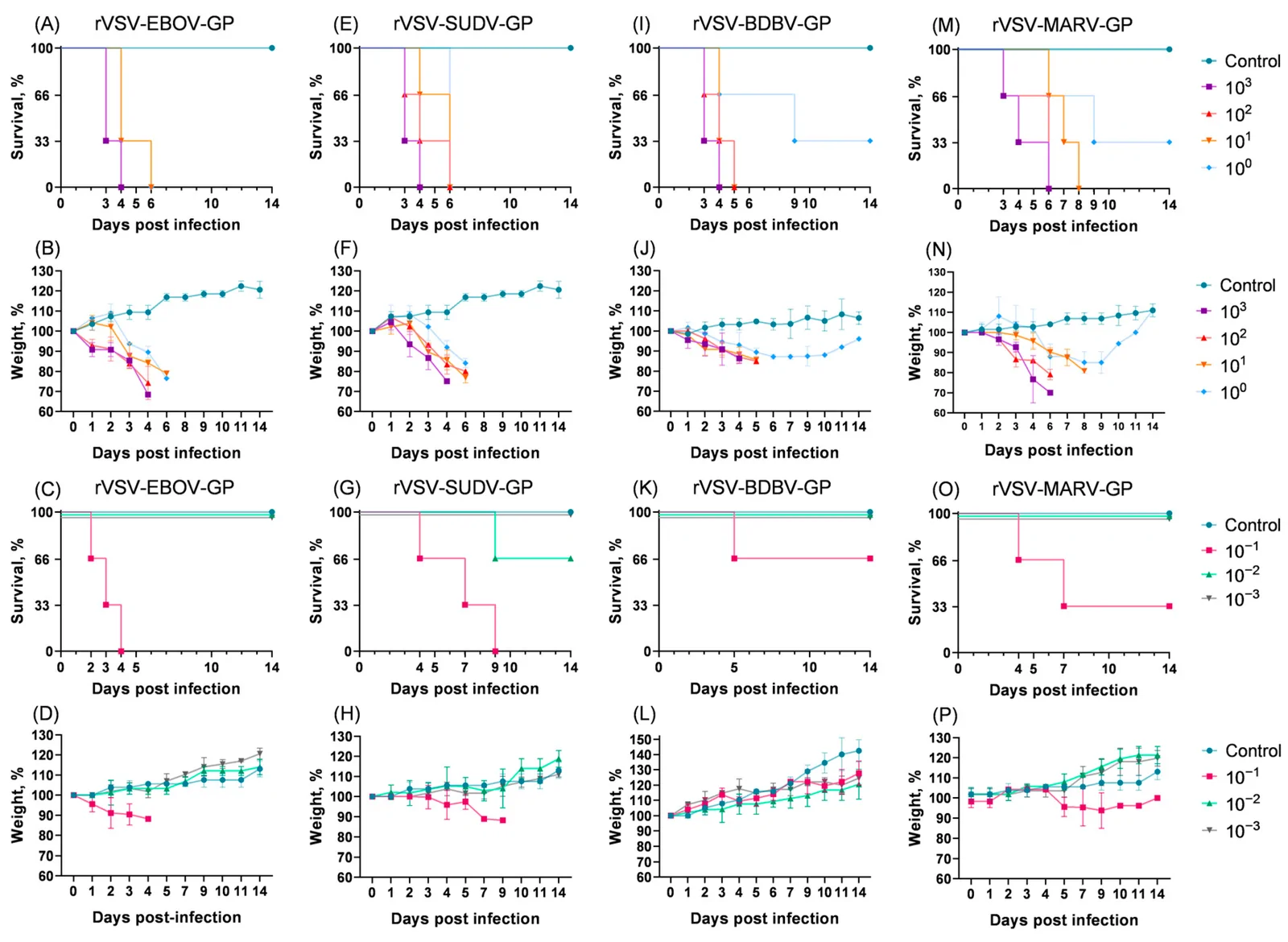
The susceptibility of *Ifnar1*-knockout mice to rVSV-Filo-GP intraperitoneal infection with varying doses. (**A**–**D**) rVSV-EBOV-GP; (**E**–**H**) rVSV-SUDV-GP; (**I**–**L**) rVSV-BDBV-GP; (**M**–**P**) rVSV-MARV-GP. The animals (n = 3) were i.p. infected with a range of doses of rVSV-Filo-GP and monitored for weight loss and survival. The control group (n = 3) was i.p. given the same volume of sterile isotonic saline solution. Weight loss was measured as the percent weight loss compared with the initial weight on day 0. The symbols of the weight graphs represent mean body weight in the groups, the bars show SD of the mean.

Next, we assessed the median lethal doses for rVSV-Filo-GP in small groups of animals. The *Ifnar1*(-/-) mice (n = 3 in one group) were i.p. infected with the rVSVs at a tenfold range of doses: 10^6^ to 10^−3^ PFU per animal. Then body weight and survival were monitored for two weeks. Death of the animals occurred at days 3–9 post-infection (Figure 3). All rVSV-EBOV-GP-infected animals in the groups 10^6^ to 10^−1^ died by the 6 dpi, all animals in the 10^−2^ and 10^−3^ groups survived. The *Ifnar1*-knockout mice infected with 10^3^ to 10^0^ PFU of rVSV-SUDV-GP also showed 100% lethality up to day 6 post-infection. The group 10^−1^ rVSV-SUDV-GP showed relatively slower decline in survival; deaths of the animals (3/3) occurred at 4–9 dpi; also, one animal (33%) succumbed in the 10^−2^ rVSV-SUDV-GP. Among the rVSV-MARV-GP- and rVSV-BDBV-GP-infected groups, 100% lethality was observed in 10^3^ to 10^1^ groups (3–8 dpi), and 66% lethality (2/3 animals) by 9 dpi in 100 groups; in 10^−1^ groups, survival rate was 33% and 66%, respectively. All infected animals showed clinical signs of the infection: hunched posture, ruffled fur and weight loss (Figure 3), but the symptoms had resolved in survivor animals by the end of the experiment. The results for the groups 10^6^, 10^5^, 10^4^ PFU per animal are not shown; survival dynamics was similar to corresponding groups with 10^3^ PFU per animal.

According to the survival data, mean lethal doses (MLDs) of rVSVs in *Ifnar1*(-/-) mice were estimated using Spearman–Karber method: rVSV-EBOV-GP—0.07 PFU per animal, rVSV-SUDV-GP—0.05 PFU per animal, rVSV-BDBV-GP—6.9 PFU per animal, rVSV-MARV-GP—0.89 PFU per animal. The MLD of rVSV-BDBV-GP was notably higher than of rVSV-MARV-GP, despite statistically identical viral loads and similar survival kinetics for rVSV-BDBV-GP.

### 3.3. Viral Load Dynamics in Ifnar1-Knockout Mice After rVSV-EBOV-GP i.p. Infection

To study viral load dynamics at 2 h, 24 h, and 48 h post-infection (hpi), the groups of *Ifnar1*-knockout mice (n = 5 for each time point) were i.p. infected with 10^4^ PFU of rVSV-EBOV-GP and the control group (n = 3) was i.p. given the same volume of sterile isotonic solution. Then the animals were euthanized at designated time points, and organs were obtained, homogenized and used for viral load assay.

**Figure 4:**
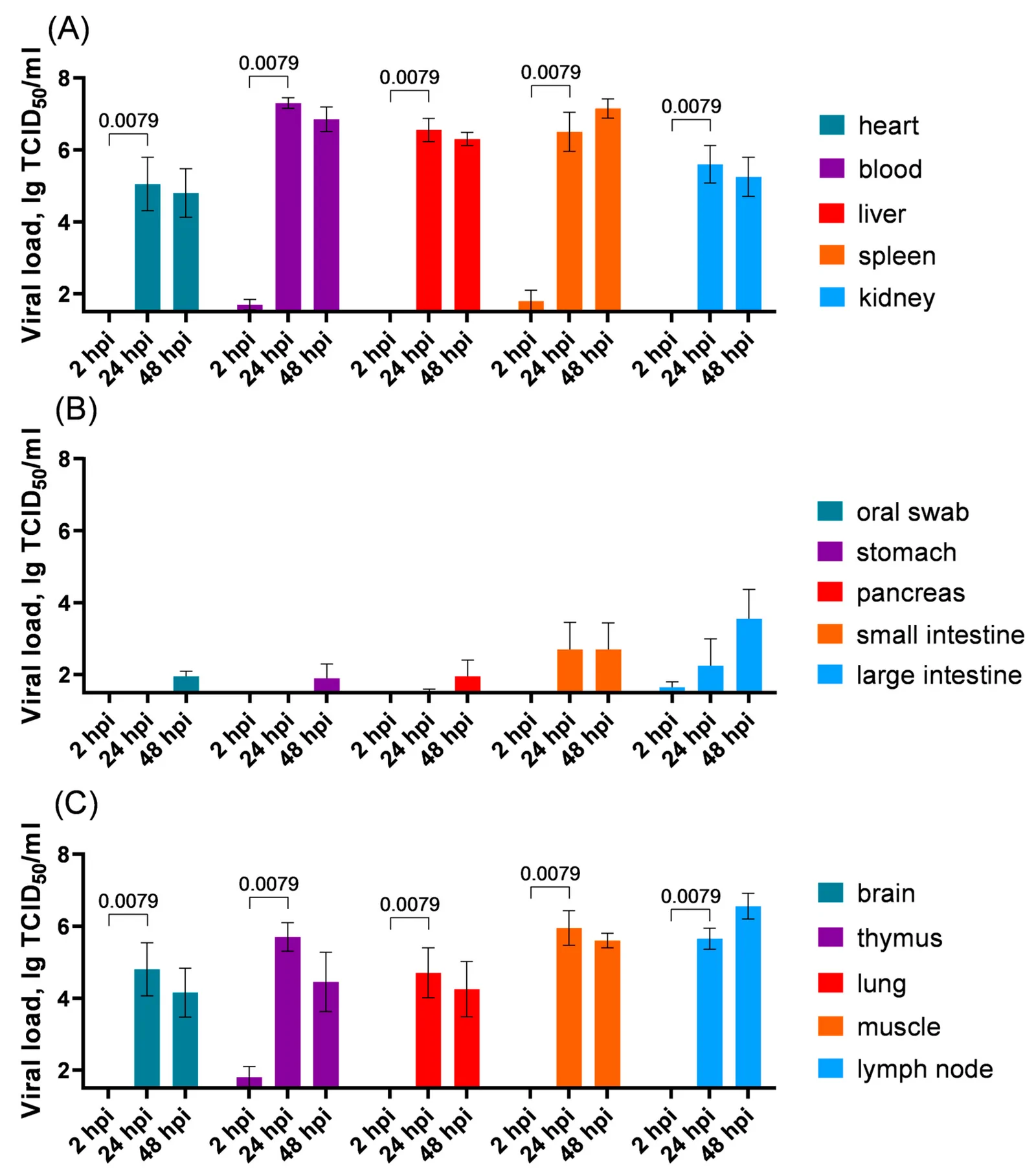
Dynamics of mean viral load in organs *Ifnar1*-knockout mice after intraperitoneal rVSV-EBOV-GP infection. The study organ groups were distributed in the following manner: (**A**) circulatory (heart, blood, liver, spleen, kidney); (**B**) gastrointestinal (oral swab, stomach, pancreas, small intestine, large intestine); (**C**) other (brain, thymus, lung, muscle, lymph node). The infectious dose was 10^4^ PFU per animal, then animals were euthanized at 2 h, 24 h, or 48 h post-infection (n = 5 at each time point) to obtain the organs. No detectable viral load was observed in control mice (n = 3). Viral load in organs is represented by TCID_50_/mL (for solid organs—TCID_50_/mL in 10% homogenates); the bars show standard error of the mean; hpi—hours post-infection. Calculated *p*-values are given above horizontal bars. Statistical significance was set at *p* < 0.0167 after Bonferroni correction for three comparisons.

The *Ifnar1*(-/-) mice, infected with rVSV-EBOV-GP, experienced significant accumulation of rVSV in organs and tissues during 48 h post-infection. Statistical analysis of post-infection viral load demonstrated that rVSV-EBOV-GP rapidly propagated (*p* = 0.0079) within the first 24 h in the most of the investigated (10 of 15) organs: blood-related (heart, blood, liver, spleen, kidney) and also in brain, thymus, lung, muscle, and lymph node samples (Figure 4). The most prominent viral load increase was detected in the blood samples: the difference between 24 h and 2 h post-infection constituted as much as 5.6 lg TCID_50_/mL. In the group of gastrointestinal organs, no significant increase in rVSV titers was detected within the first 24 hpi in all investigated organs (oral swab, stomach, pancreas, small intestine, large intestine). As for 24–48 hpi period, viral titers did not significantly increase. No detectable viral load was observed in all samples of the control animals (n = 3).

### 3.4. Viral Load in Ifnar1-Knockout Mice After i.p. rVSV-Filo-GP Infection

Then we investigated viral load of filoviral GP-pseudotyped rVSVs in organs of *Ifnar1*(-/-) mice 48 h post-infection (hpi). Groups of animals (n = 5) were i.p. infected with 10^4^ PFU of rVSV-SUDV-GP, rVSV-BDBV-GP, or rVSV-MARV-GP and the control group (n = 3) was i.p. given sterile isotonic solution. Then the animals were euthanized at 48 hpi, and the organs were obtained, homogenized and used for viral load assay.

**Figure 5:**
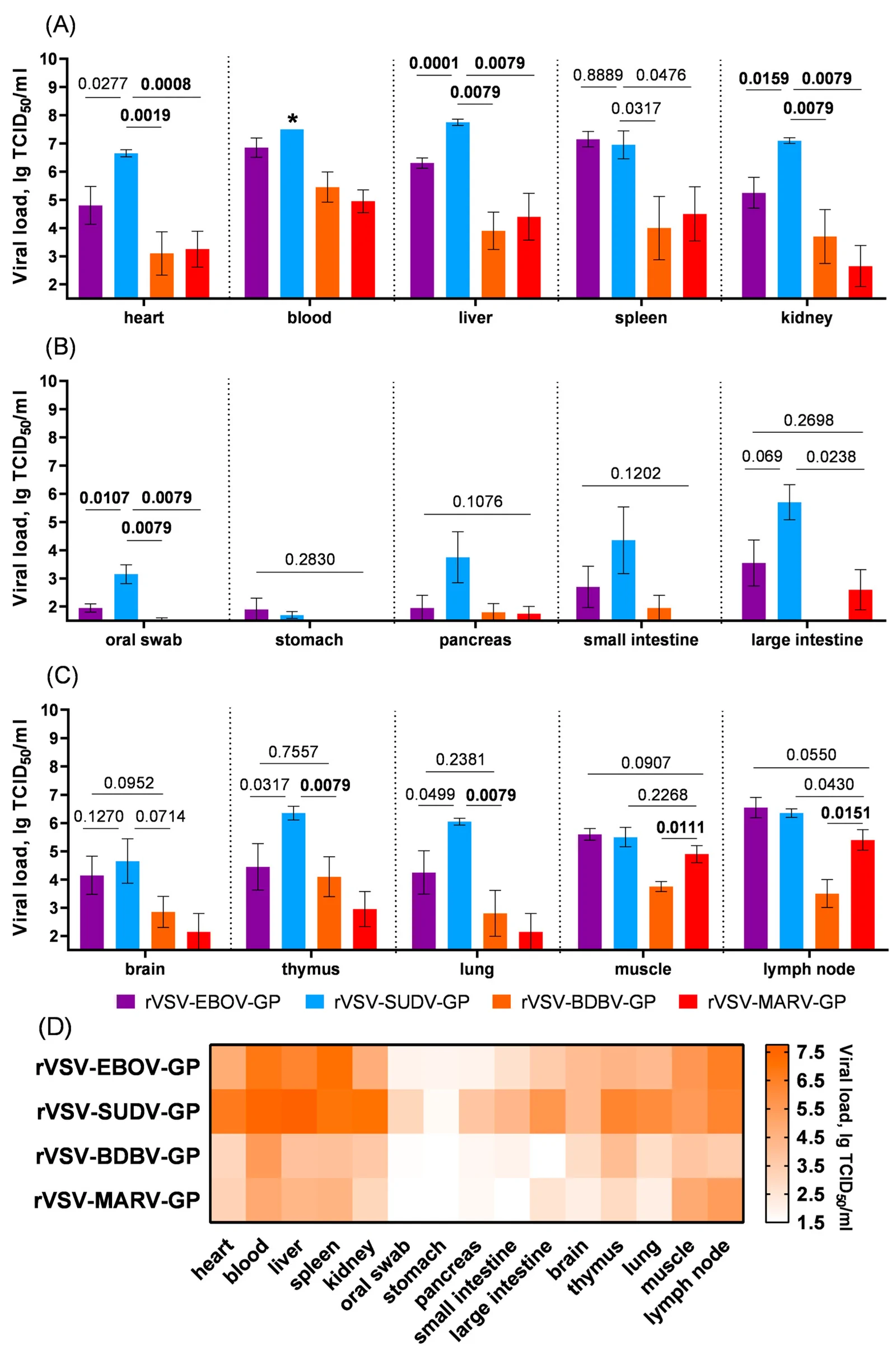
Mean viral load in organs of *Ifnar1*-knockout mice 48 h post-infection (hpi) with rVSV-Filo-GP. Groups of organs: (**A**) circulatory (heart, blood, liver, spleen, kidney); (**B**) gastrointestinal (oral swab, stomach, pancreas, small intestine, large intestine); (**C**) other (brain, thymus, lung, muscle, lymph node); (**D**) heat map of mean viral load. The mice were i.p. infected with 10^4^ PFU of rVSV-Filo-GP (n = 5 for each vector) and euthanized at 48 hpi to obtain the organs. No detectable viral load was observed in control mice (n = 3). Viral load in organs is represented by TCID_50_/mL (for solid organs—TCID_50_/mL in 10% homogenates); the bars show standard error of the mean. *p*-values are displayed above the horizontal bars, and statistically significant values are indicated in bold. Statistical significance was set at *p* < 0.0167 after Bonferroni correction for three comparisons. * Error bars are not shown for the blue bar in panel blood, Subfigure (**A**), because the value of 7.5 lg TCID50/mL was obtained in all five samples, as the upper limit of detection. However, we were not able to subsequently repeat viral load analysis could for these blood samples because of the limited amount of material available. Because this group represents a single value at the upper limit of detection, statistical comparison for this group was not performed.

No significant difference between all rVSV-infected groups was detected in stomach (*p* = 0.2830), pancreas (*p* = 0.1076) or small intestine (*p* = 0.1202) due to the low viral load (Figure 5). The highest viral load was detected in the rVSV-SUDV-GP-infected group in the majority of the organs (11 of 15). It was significantly higher compared to all other groups in oral swab, liver and kidney. The highest values of viral load in the spleen, stomach, muscle and lymph node were detected in the rVSV-EBOV-GP-infected group, but they did not differ significantly from the values in the rVSV-SUDV-GP group.

The rVSV-BDBV-GP- and rVSV-MARV-GP-infected groups showed major concurrence in rVSV propagation: no significant difference in viral titers was detected in 13 of 15 organs. Significantly higher titers were observed in the muscle (*p* = 0.0111) and lymph node (*p* = 0.0151) in the rVSV-MARV-GP-infected group. In total, rVSV-SUDV-GP group showed the most prominent mean viral load of 5.52 lg TCID_50_/mL across all organs, while for the rVSV-EBOV-GP-infected group it was 4.41 lg; rVSV-BDBV-GP and rVSV-MARV-GP both showed relatively lower overall mean viral loads of 2.98 and 3.06 lg, respectively.

**Figure 6:**
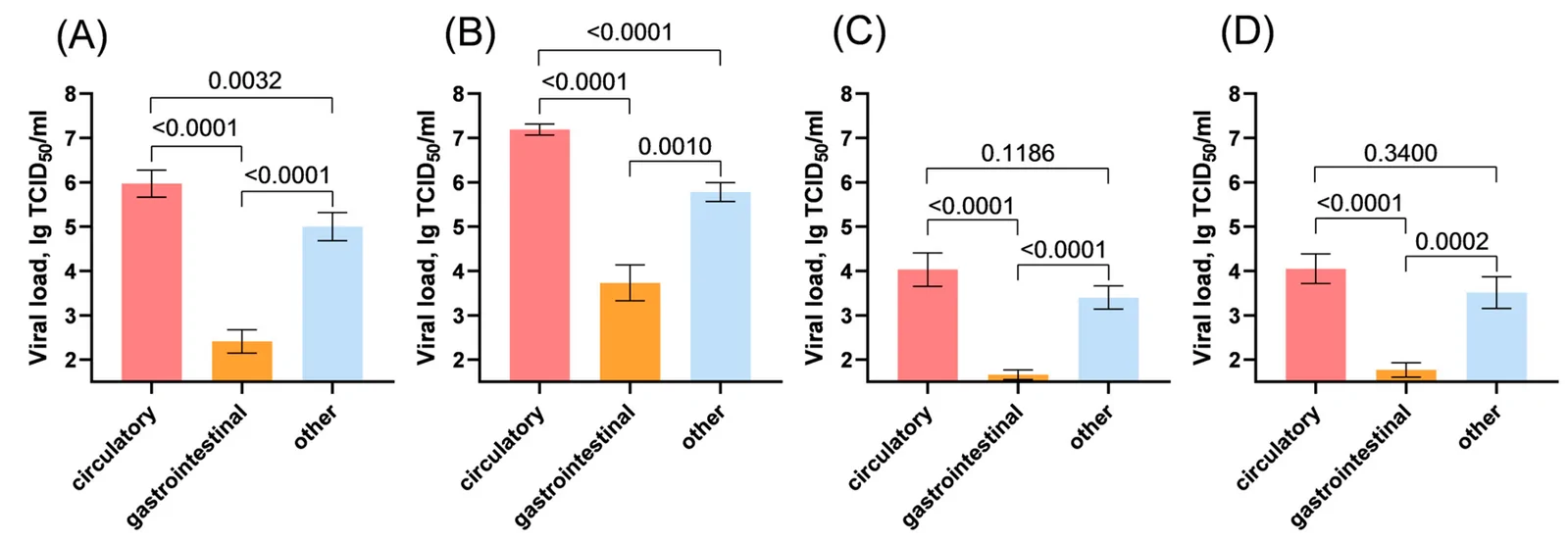
Mean viral loads in groups of organs of *Ifnar1*-knockout mice 48 h post-infection (hpi) with rVSV-Filo-GP. (**A**) rVSV-EBOV-GP; (**B**) rVSV-SUDV-GP; (**C**) rVSV-BDBV-GP; (**D**) rVSV-MARV-GP. The mice (n = 5) were i.p. infected with 10^4^ PFU of rVSV-Filo-GP and euthanized at 48 hpi to obtain the organs. The study organ groups were distributed in the following manner: circulatory (heart, blood, liver, spleen, kidney); gastrointestinal (oral swab, stomach, pancreas, small intestine, large intestine); other (brain, thymus, lung, muscle, lymph node). No detectable viral load was observed in control mice (n = 3). Viral in organs is represented by TCID_50_/mL (for solid organs—TCID_50_/mL in 10% homogenates); the bars show standard error of the mean. Viral load data were statistically analyzed with Mann–Whitney test. *p*-values are displayed above the horizontal bars, and statistically significant values are indicated in bold. Statistical significance was set at *p* < 0.0167 after Bonferroni correction for three comparisons.

To obtain a more generalized overview of rVSV-Filo-GP distribution in the organs of *Ifnar1*-knockout mice, we compared the viral loads in three groups of organs: circulatory (heart, blood, liver, spleen, kidney); gastrointestinal (oral swab, stomach, pancreas, small intestine, large intestine); other (brain, thymus, lung, muscle, lymph node). The samples in the gastrointestinal group showed significantly lower titers in general for all rVSVs compared to the circulatory (*p* < 0.0001) and the other (*p* ≤ 0.0010) groups of organs (Figure 6). For rVSV-EBOV-GP and rVSV-SUDV-GP, significantly higher viral loads were observed in organs connected with the circulatory system or with an extensive blood supply (*p* = 0.0032 and *p* < 0.0001, respectively) in rVSV-BDBV-GP- and rVSV-MARV-GP-infected animals. In contrast to this, the viral load in the circulatory group did not significantly differ from the other group (*p* = 0.1186 and *p* = 0.3400, respectively) for both rVSV-MARV-GP and rVSV-BDBV-GP, as they demonstrated relatively more prominent viral load in the thymus (rVSV-BDBV-GP), lymph node (rVSV-MARV-GP) and muscle tissue (both).

## 4. Discussion

To improve accessibility in filovirus vaccine research, BSL-2-compatible surrogate rVSV-based animal models have been recently developed, using Syrian hamsters and transgenic mice. Previous studies have shown that Syrian hamsters demonstrate limited ability to model filoviral infections using recombinant VSV. Only high dose of rVSV-EBOV (10^6^ PFU) was 100% lethal and rVSV-MARV was less pathogenic even at a higher dose: 10^7.5^ PFU—33% survival [15]. In another study, 100% of Syrian hamsters survived after 10^7^ TCID_50_ i.p. inoculation of VSV-SUDV-GP [16]. A model of EBOV infection was also established in type I and II Interferon receptors-knockout IFNα/β/γ R^–/–^ mice and showed similar survival dynamics: 100% of animals succumbed at 3 dpi in both dose groups (10^5^ and 10^6^ PFU) after i.p. inoculation of rVSVΔG-EBOV/GP, but alternative routes were not tested [17]. At the same time, among the Syrian hamsters, infected with 10^7^ TCID_50_ VSV-EBOV/GP via intraperitoneal subcutaneous, intramuscular and intranasal routes, only the i.p. route resulted in lethal disease, which animals succumbed to at 3 dpi [16]. Likewise, Syrian hamsters i.p. infected with 10^7.2^ PFU of rVSV/EBOV succumbed at 3 dpi [15]. Also, an exotic route for rVSV-EBOV was tested in the study focused on neurovirulence assessment of chimeric vaccine (PHV02) against Ebola and Nipah viruses: Swiss-Webster mice were intracranially infected with rVSV-EBOV and showed no clinical signs of the infection or specific death during 21 days post-infection [18]. A lethal challenge of *Ifnar1*(-/-) mice with 100 PFU VSV-SUDV consistently induced rapid weight loss, progressive clinical deterioration, and death by 3–4 dpi. The resulting infection was accompanied by thrombocytopenia and leukopenia, particularly lymphopenia, consistent with systemic viral infection and consumptive coagulopathy [19]. Together, these findings established surrogate models for studying GP-targeting therapeutic strategies under BSL-2 conditions.

Since the developed models are focused on EBOV infection, in our study we aimed to extend BSL-2-compatible models to other filoviruses (SUDV, BDBV, MARV) using pseudotyped recombinant VSVs and to provide detailed characterization of these models to enhance reproducibility in animal studies. First, we showed that *Ifnar1*(-/-) transgenic mice were highly susceptible to rVSV-Filo-GP infection even after non-natural exposure routes, as intramuscular and intranasal inoculation also resulted in 100% lethality, similar to intraperitoneal route.

Variability of estimated mean lethal doses (MLDs) in our study is consistent with difference in lethality of filoviruses; however, the obtained MLD values are approximate due to a small sample size (n = 3) and need validation in experiments with greater sample size. According to the recent meta-analyses of global outbreak data, pooled case fatality rates were estimated as following: 32.8% for BDBV, 48.5% for SUDV, 66.6% for EBOV [20], and 61.9% for MARV [21]. Although the obtained values of median lethal doses of rVSVs are low and not intuitively expected (<1 PFU per animal), they are consistent with methodological parameters of plaque assay: the accuracy range of ±0.5 lg PFU/mL and CV of 30% account for biological variability in in vitro assays, confirmed with validated plaque assay protocols [22]. Considering differences in rVSV genetic sequences between different studies, titration methods, plaque assay format, and experimental design, the obtained estimated mean lethal doses mainly reflect the relative differences between rVSV-Filo-GP vectors within a described framework. Nevertheless, a low lethal dose of wild-type EBOV was also shown in Cynomolgus macaques: 100% of the primates died after high-particle/PFU-ratio infection with 0.01 PFU per animal [23]. Recently established lethal VSV-based surrogate mouse models for Sudan and Bundibugyo ebolaviruses in *Ifnar1*(-/-) mice reported low LD_50_ values for VSVΔG-SUDV GP and VSVΔG-BDBV GP (1.027 and 1.000 PFU, respectively), consistent with our data [24].

**Figure A2:**
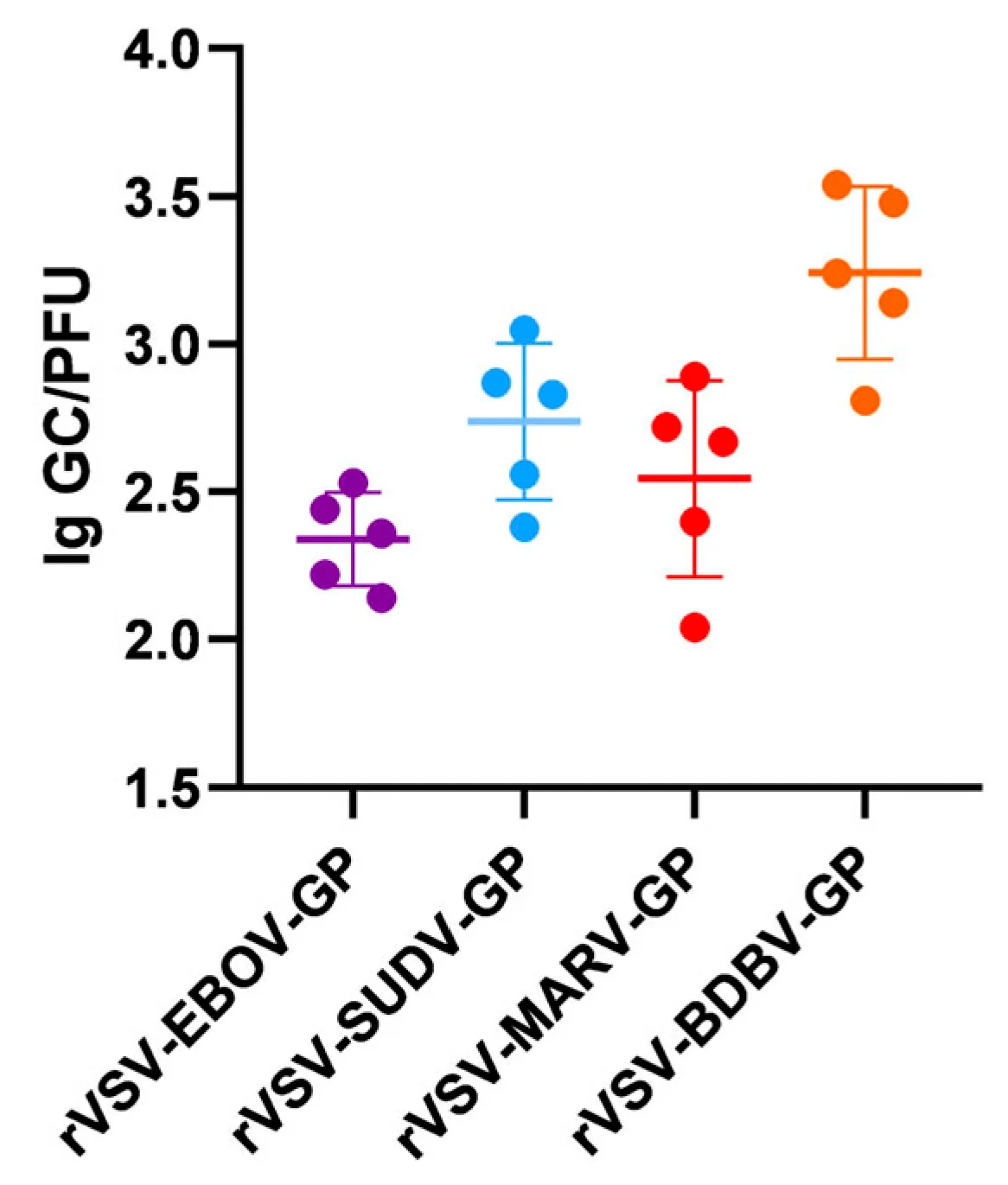
GC/PFU ratio of rVSV-Filo-GP batches, n = 5 for each vector. Coefficient of GC/PFU ratio variation: rVSV-EBOV-GP—6.792%, rVSV-SUDV-GP—9.716%, rVSV-MARV-GP—13.06%, rVSV-BDBV-GP—9.026%.

According to PCR results or rVSV-Filo-GP batches used for inoculation (Figure A2), rVSV-BDBV-GP showed the highest ratio of GC to PFU. Defective infectious particles (DIPs) contribute to high genome counts, and compete with standard infectious virions for replication machinery and packaging, possibly reducing the yield of infectious progeny, and thereby attenuating infection [25]. The GC/PFU ratios are consistent with estimated mean lethal doses, suggesting that relatively higher DIP content in rVSV-BDBV-GP could possibly account for higher mean lethal dose. However, profiling of DVG (defective viral genomes) is needed to confirm this hypothesis, which was beyond the scope of the present study.

While comparable peak viral loads of rVSV-BDBV-GP and rVSV-MARV-GP could be attributed to rVSV-backbone effects, differences in MLD between rVSV-BDBV-GP and rVSV-MARV-GP could reflect GP-mediated variability in cell entry efficiency, or GP-mediated interactions with target tissues, which contribute to variability in pathogenesis and lethality of filoviruses. Recombinant VSV pseudotyped with filoviral GP represents a valid pseudotype virus system, that has been established and used for functional analysis of filovirus GPs, including tropism [26]. Filoviral glycoproteins have been reported to vary in entry characteristics, tissue tropism and direct pathogenic effects in vitro and in vivo in a number of distinct studies. Wild-type BDBV has shown reduced virus yields and cytokine production in human PBMCs, compared to EBOV [27]. Recent study of the in vivo pathogenic effect of MARV GP has shown that MARV GP increases vascular permeability and exacerbates inflammation and tissue injury in mouse muscle and liver transduction models [28]. Animal studies with strict side-by-side experimental design to compare pathogenic effects of rVSV-Filo-GP with wild-type filoviruses, as well as various wild-type filoviruses (including MARV vs. BDBV), are yet to be established.

Regarding viral load data, a direct comparison with data in other studies is not possible due to variability in mouse models or infectious doses: for *Ifnar*-/- mice, an infectious dose of 100 PFU was used [29]; in another study for IFNα/β/γ R^–/–^ mice, infectious doses of 1 × 10^5^ and 1 × 10^6^ PFU were used [17]. Filoviruses exhibit pantropism when infecting primates, with preference for hepatocytes, endothelial cells, dendritic cells, monocytes, and macrophages [26]. In this study, high viral load was detected in liver and immune cell reservoirs (blood, spleen, thymus and lymph node), supporting systemic dissemination in the experimental model. Our findings represent the total recoverable viral load from the organ homogenate, rather than a virus strictly confined to a blood-free parenchymal compartment.

**Figure A3:**
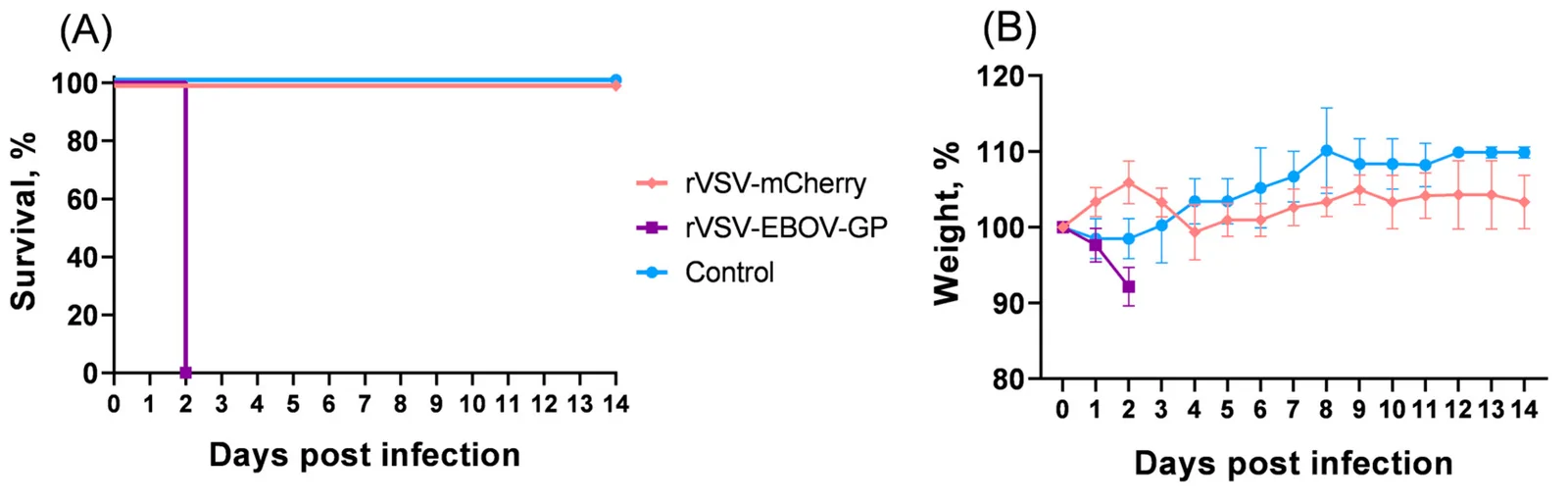
GP-mediated lethality of rVSV-EBOV-GP in male *Ifnar1*-knockout mice. (**A**) Survival, (**B**) weight of the mice. The animals were i.p. inoculated with 3.5 lg TCID50 of rVSV-EBOV-GP (n = 5), 3.5 lg TU of rVSV-mCherry (n = 5), or 0.5 mL of PBS (n = 3) as a control group. The animals were daily monitored for weight changes and survival for 14 days. All animals in rVSV-EBOV-GP group have succumbed at 2 dpi. All animals in the control and rVSV-mCherry groups have survived and did not show clinical symptoms of infection.

Previous studies have shown that VSVΔG lacking an envelope glycoprotein does not disseminate efficiently in tissues and organs in vivo [30,31]. To further examine the contribution of GP-mediated entry to the pathogenic effect of rVSV-Filo-GP in *Ifnar1*(-/-) mice, we inoculated *Ifnar1*(-/-) mice intraperitoneally with rVSV-EBOV-GP or rVSV(ΔG)-mCherry. As a result, rVSV(ΔG)-mCherry did not cause clinical symptoms, or lethal effect in mice, as well as detectable accumulation of recombinant virus in blood, which is in line with the expected entry limitations of glycoprotein-deficient VSV particles (Figure A3). These findings support our view that GP-mediated entry is an important determinant of the observed phenotype. However, quantification of entry efficiency and direct tissue-level detection of GP transgene expression in vivo were beyond the scope of the present study.

Considering the influence of rVSV backbone, the rVSV-Filo-GP vectors could be expected to have very similar growth kinetics, conditioned by the same polymerase and protein composition among all vectors. However, this was not the case in our study. On the contrary, we observed delayed lethality and lower peak titers in rVSV-BDBV-GP, compared to rVSV-EBOV-GP and rVSV-SUDV-GP, which is consistent with in vitro and non-human primate (NHP) data [32]. Pathogenesis of wild-type EBOV and SUDV was extensively studied in NHPs and the collected evidence highlights minor differences between these filoviruses [33]. In contrast to NHP data, peak viral loads of rVSV-SUDV-GP in *Ifnar1*(-/-) mice were considerably greater than of rVSV-EBOV-GP, but not in in vitro conditions, in Vero E6 cells. Interestingly, this was in accordance with other mouse models: wild-type SUDV demonstrated greater lethality than EBOV at equivalent doses in STAT1-KO mice [34]. Alignment of rVSV and wild-type filoviruses data in transgenic mouse models suggests that SUDV-GP interaction with host immune cells in conditions of dysregulated immune pathways could provide some advantage in dissemination or replication of SUDV.

To our knowledge, this is the first work that establishes and characterizes lethal model of GP-mediated entry of rVSV-MARV-GP in *Ifnar1*-knockout mice. The surrogate rVSV-EBOV, rVSV-SUDV, and rVSV-BDBV models in *Ifnar*-/- mice were described previously [19,24,26], although some of the studies focused primarily on vaccine characterization or immune responses. Surrogate lethal rVSV-based models were also described in other animals: type I and II Interferon receptors-knockout (IFNα/β/γ R^–/–^) mice [17] and Syrian hamsters [15,16]. Mouse models are commonly based on intraperitoneal administration, which was also used in this study; however, we additionally demonstrated lethality in *Ifnar1*(-/-) mice following rVSV inoculation via multiple routes, including i.m., i.n., and gastric administration, underscoring the need to evaluate different exposure routes, given the reported variability in disease progression and lethality in nonhuman primates [12].

Beyond Ebola virus disease, small-animal infection models for Sudan ebolavirus, Bundibugyo ebolavirus, and Marburg virus remain scarce, thereby impeding research progress; the present study addresses this critical gap and provides additional data that complement previous findings and facilitate. However, the developed models need further clinical characterization, such as histological assessment, investigation of blood parameters and immune response [12]. *Ifnar1*-knockout mice are not suitable for direct immunization with rVSV-based filoviral vaccines due to their pathogenic effect in these immunocompromised animals, but this allows for studying adoptive immune response (transfer of sera or immune cells from other animals). In spite of this, mice are considered more convenient models for immunological studies than other species of rodents, due to more abundant reagents [12].

A major driver of lethality in immunocompromised *Ifnar1*(-/-) mice is uncontrolled replication of the rVSV vector; therefore, we used this model to evaluate subsequent in vivo replication and acute lethality under conditions of compromised type I IFN signaling, rather than a model that represents filoviral pathogenesis. Filoviral pathogenesis is a systemic and multifactorial process characterized by widespread viral dissemination, dysregulated innate and adaptive immune responses, vascular dysfunction, and multi-organ injury. Clinical manifestations of filoviral infection include macrophage/DC dysregulation phenotypes, classical DIC/coagulopathy, pronounced lymphocyte apoptosis, characteristic hepatic lesions, etc. [6,12,14]. Surrogate rVSV infections do not fully recapitulate filoviral pathogenesis, but prior studies showed that rVSV models can reproduce distinct features of filoviral pathogenesis: rVSV/EBOV and rVSV/MARV in hamsters caused liver and spleen pathology, infection of monocytic cells, thrombocytopenia, and elevated liver enzymes [15]; hamsters infected with VSV-EBOV developed hyperviremia and distinct splenohepatic lesions [16], severe liver and spleen lesions in IFNα/β/γR^–/–^ mice [17]. Recombinant viral vectors offer a highly promising strategy for establishing surrogate models of viral infections. Earlier, HIV-based pseudoviruses were used to model Ebola virus, Marburg virus, and Lloviu virus infections in BALB/c mice for filovirus entry inhibitors evaluation [35]. Recombinant VSVs (rVSV-SARS2-S) were used to create BSL-2 compatible model of SARS-CoV-2 infection in K18-ACE2 mice [36]. Type 1 IFN-receptor knock-out (*Ifnar*-/-) mouse model was employed for challenge studies of Zika virus vaccine candidate [37].

In the present study, we showed that *Ifnar1*-knockout mice infection with recombinant VSVs, pseudotyped with filoviral GPs and carrying corresponding GP gene sequences, allowed to model lethal EBOV-, SUDV-, BDBV- and MARV- GP-mediated entry in BSL-2 conditions to provide safe and effective in vivo research of GP-targeted filoviral vaccines. This allows to perform broad-scale pilot studies of GP-targeted vaccines using surrogate challenge that models filoviral GP-mediated entry. Commercially available transgenic *Ifnar1*(-/-) mice enable broad adoption of the model, thereby accelerating progress in the field. Further development of accessible recombinant virus-based animal models for BSL-4 pathogens could enhance efficacy studies of vaccines and therapeutic antibodies.

## Figures and Tables

**Figure A1:**
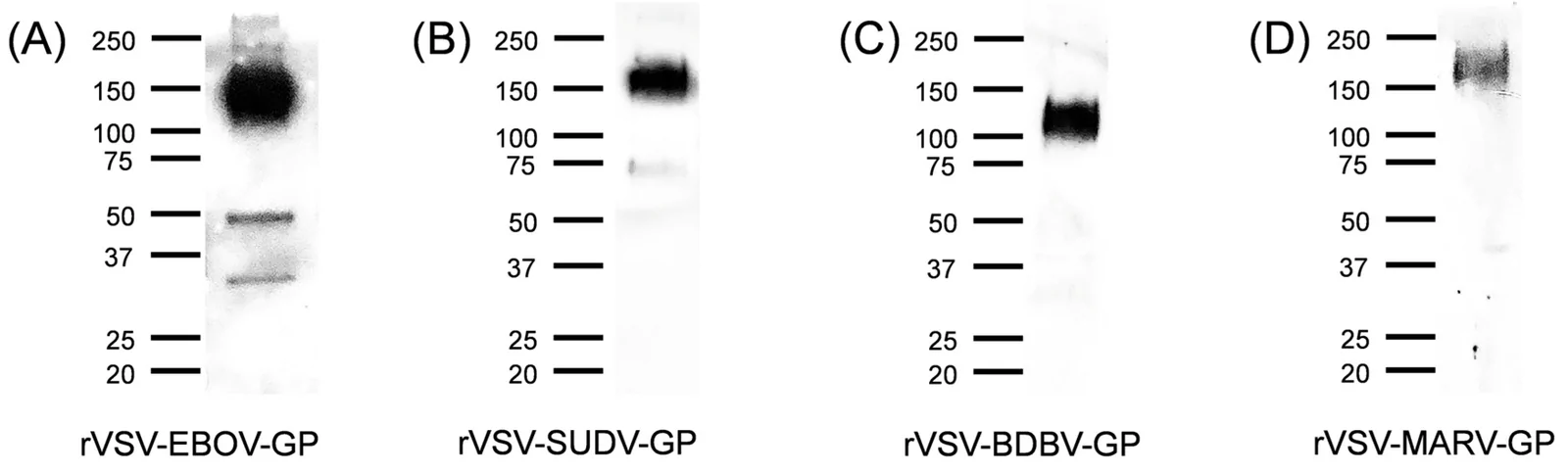
Western blot of conditioned media 48 h after rVSV-Filo-GP inoculation of Vero E6 cells at MOI = 0.01: (**A**) rVSV-EBOV-GP, (**B**) rVSV-SUDV-GP, (**C**) rVSV-BDBV-GP, (**D**) rVSV-MARV-GP.

**Figure A4:**
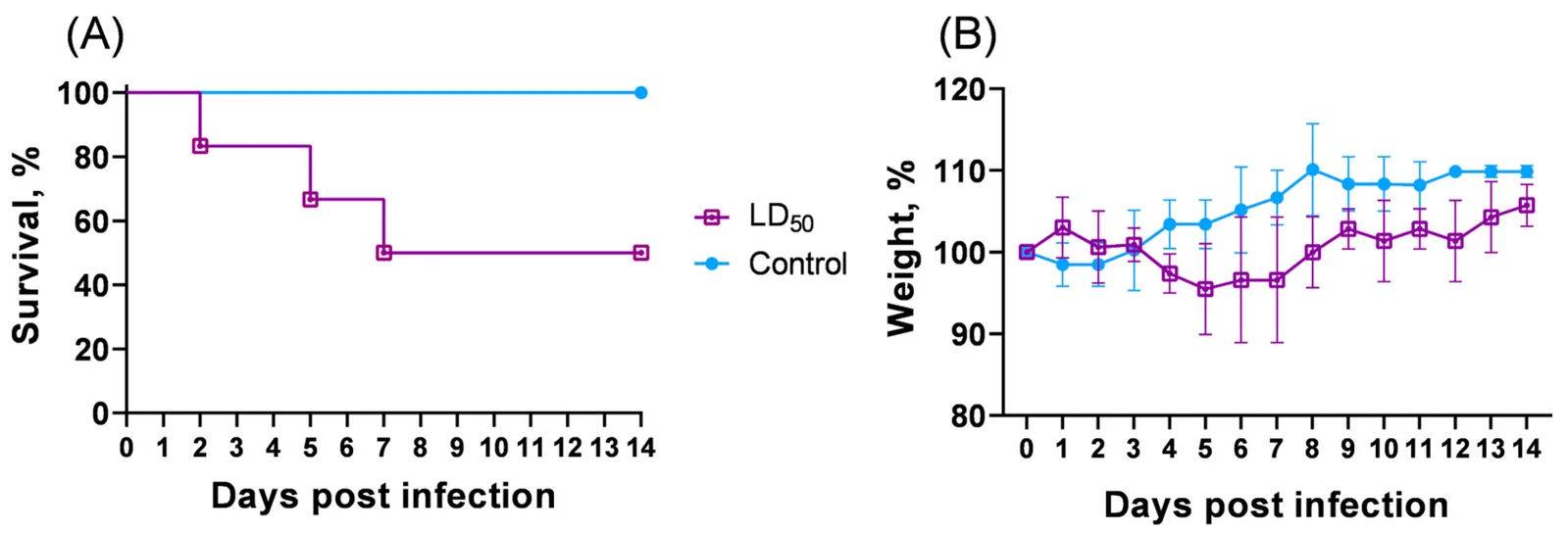
Lethality assessment for mean lethal dose of rVSV-EBOV-GP in male *Ifnar1*-knockout mice. (**A**) Survival, (**B**) weight of the mice. The animals were i.p. given 0.07 PFU of rVSV-EBOV-GP (n = 6), or 0.5 mL of PBS (n = 3) and were daily monitored for weight changes and survival for 14 days. 3/6 animals in rVSV-EBOV-GP group have succumbed by the end of the experiment (2, 5, 7 dpi). All animals in the control group have survived and did not show clinical symptoms of infection.

**Figure A5:**
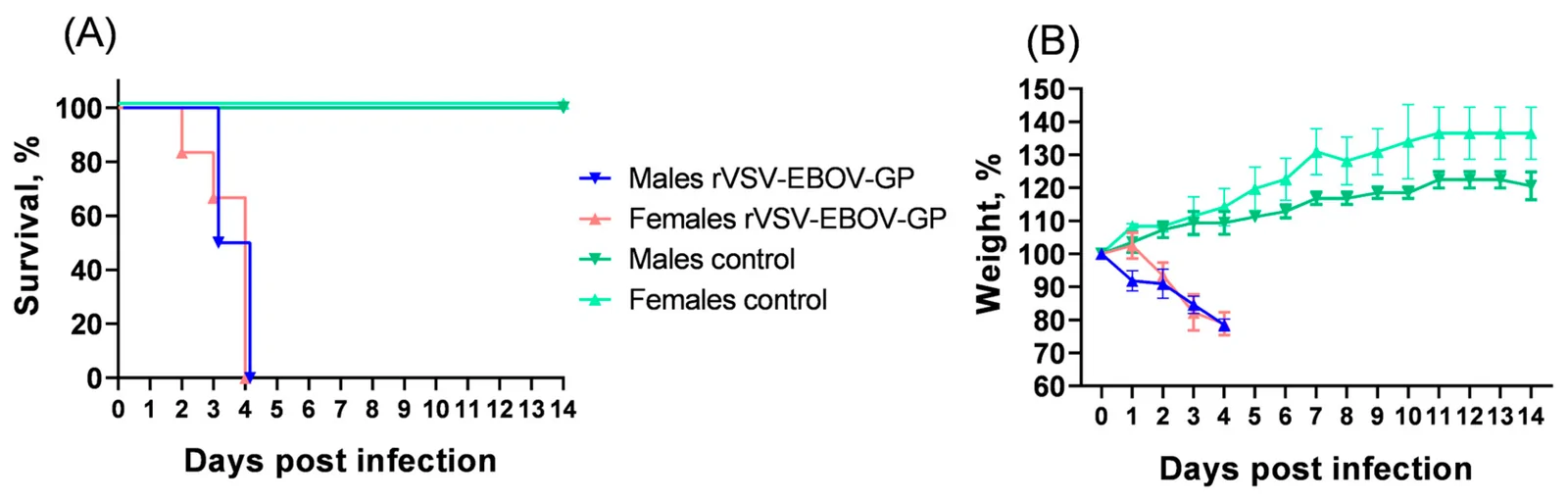
Lethality of rVSV-EBOV-GP in male and female *Ifnar1*-knockout mice. (**A**) Survival, (**B**) weight of the mice. Both male and female animals were i.p. given 10^4^ PFU of rVSV-EBOV-GP (*n* = 6), or 0.5 mL of PBS (*n* = 3) and were daily monitored for weight changes and survival for 14 days. Median survival time in rVSV-EBOV-GP groups was 3 days for male mice and 4 days for female mice. Survival curves were generated using the Kaplan–Meier method and compared by the log-rank (Mantel–Cox) test (χ2 = 0.107, *p* = 0.743).

## Data Availability

The data presented in this study are available on reasonable request from the corresponding author.

## Abbreviations

The following abbreviations are used in this manuscript:

rVSV	recombinant vesicular stomatitis virus
EBOV	Ebola virus
SUDV	Sudan virus
BDBV	Bundibugyo virus
MARV	Marburg virus

## Appendix A

**Table A1:** rVSV-Filo-GP growth kinetics in Vero E6 cells.

Viral Titer, PFU/mL	rVSV-EBOV-GP	rVSV-SUDV-GP	rVSV-BDBV-GP	rVSV-MARV-GP
24 hpi	3.1 × 10^7^ ± 5.0 × 10^6^	1.5 × 10^7^ ± 3.7 × 10^6^	3.6 × 10^6^ ± 7.9 × 10^5^	1.2 × 10^7^ ± 2.2 × 10^6^
48 hpi	2.7 × 10^7^ ± 2.2 × 10^6^	1.5 × 10^7^ ± 4.0 × 10^6^	3.9 × 10^6^ ± 8.5 × 10^5^	2.1 × 10^7^ ± 6.0 × 10^6^
72 hpi	1.2 × 10^7^ ± 1.9 × 10^6^	6.6 × 10^6^ ± 2.8 × 10^6^	1.8 × 10^6^ ± 2.0 × 10^5^	1.3 × 10^7^ ± 4.0 × 10^6^
96 hpi	1.2 × 10^7^ ± 9.3 × 10^6^	2.8 × 10^6^ ± 7.0 × 10^5^	9.8 × 10^5^ ± 7.3 × 10^4^	8.4 × 10^6^ ± 1.5 × 10^6^

**Table A2:** rVSV-Filo-GP plaque size at 48 h post-infection (0.01 MOI) of Vero E6 cells.

Plaque Size, mm^2^	rVSV-EBOV-GP	rVSV-SUDV-GP	rVSV-BDBV-GP	rVSV-MARV-GP
Mean size	7.708	3.458	2.671	4.532
SD of the mean	1.261	0.4714	0.9746	0.7904
